# A Small Disc Area Is a Risk Factor for Visual Field Loss Progression in Primary Open-Angle Glaucoma: The Glaucoma Stereo Analysis Study

**DOI:** 10.1155/2018/8941489

**Published:** 2018-03-21

**Authors:** Yasushi Kitaoka, Masaki Tanito, Yu Yokoyama, Koji Nitta, Maki Katai, Kazuko Omodaka, Toru Nakazawa

**Affiliations:** ^1^Department of Ophthalmology, St. Marianna University School of Medicine, 2-16-1 Sugao, Miyamae-ku, Kawasaki, Kanagawa 216-8511, Japan; ^2^Division of Ophthalmology, Matsue Red Cross Hospital, 200 Horomachi, Matsue, Shimane 690-8506, Japan; ^3^Department of Ophthalmology, Shimane University Faculty of Medicine, Enya 89-1, Izumo, Shimane 693-8501, Japan; ^4^Department of Ophthalmology, Tohoku University Graduate School of Medicine, 1-1 Seiryo-machi, Aoba-ku, Sendai 980-8574, Japan; ^5^Department of Ophthalmology, Fukui-ken Saiseikai Hospital, 7-1 Funabashi, Wadanaka-machi, Fukui 918-8503, Japan; ^6^Department of Ophthalmology, Sapporo Medical Center Nippon Telegraph and Telephone East Corporation, South 1 West 15, Chuo-ku, Sapporo 060-0061, Japan

## Abstract

**Purpose:**

The Glaucoma Stereo Analysis Study, a cross-sectional multicenter collaborative study, used a stereo fundus camera (nonmyd WX) to assess various morphological parameters of the optic nerve head (ONH) in glaucoma patients. We compared the associations of each parameter between the visual field loss progression group and no-progression group.

**Methods:**

The study included 187 eyes of 187 patients with primary open-angle glaucoma or normal-tension glaucoma. We divided the mean deviation (MD) slope values of all patients into the progression group (<−0.3 dB/year) and no-progression group (≧−0.3 dB/year). ONH morphological parameters were calculated with prototype analysis software. The correlations between glaucomatous visual field progression and patient characteristics or each ONH parameter were analyzed with Spearman's rank correlation coefficient.

**Results:**

The MD slope averages in the progression group and no-progression group were −0.58 ± 0.28 dB/year and 0.05 ± 0.26 dB/year, respectively. Among disc parameters, vertical disc width (diameter), disc area, cup area, and cup volume in the progression group were significantly less than those in the no-progression group. Logistic regression analysis revealed a significant association between the visual field progression and disc area (odds ratio 0.49/mm^2^ disc area).

**Conclusion:**

A smaller disc area may be associated with more rapid glaucomatous visual field progression.

## 1. Introduction

Glaucomatous optic neuropathy (GON) is characterized by axon degeneration that can be observed as the thinning of the neural rim and enlargement of the cup in the optic nerve head (ONH). The Disc Damage Likelihood Scale (DDLS) was developed by Bayer et al. [1] and Spaeth et al. [2] for assessing the degree of optic nerve damage in GON. This method reflects the disc size and divides discs into three sizes, small (<1.5 mm), middle (1.5–2.0 mm), and large (>2.0 mm), and combines the disc size with the radial width of the neural rim or the circumferential extent of the absence of the neural rim [1, 2]. It was shown that the DDLS significantly correlates with all global and sectoral visual field indexes and with the sectoral rim area in Heidelberg Retina Tomograph (HRT) II measurements [3].

A simultaneous stereo fundus camera with analysis software (nonmyd WX, Kowa Company, Ltd., Japan) can provide ONH images in 3D and a detailed quantitative display of the ONH parameters. The Glaucoma Stereo Analysis Study (GSAS) is a multicenter study using this system to estimate various morphological parameters of the ONH in Japanese patients with GON [4, 5]. The GSAS previously demonstrated that the DDLS stage obtained through stereoscopic analysis was significantly inversely correlated with the MD and positively correlated with the pattern standard deviation (PSD) [6]. These findings suggest that the DDLS stage reflects the degree of visual field damage that is measured and expressed by the MD and PSD [6]. Since smaller discs can be categorized in higher stages in the DDLS system, and a human histological study in a relatively large number of eyes (72 eyes from 56 donors) found increasing axon numbers with greater optic disc size [7], it is reasonable to speculate that the disc size may affect glaucomatous visual field progression. In the present phase of the GSAS, we examined the relationships between visual field progression and patient characteristics or various ONH parameters.

## 2. Patients and Methods

The GSAS is a multicenter collaborative study and was conducted in accordance with the tenets of the Declaration of Helsinki. The Institutional Review Boards of the Tohoku University Graduate School of Medicine, Shimane University Faculty of Medicine, Fukui-ken Saiseikai Hospital, Sapporo Teishin Hospital, and St. Marianna University School of Medicine approved this study. The formal written consent from patients is not required for this type of study, a hospital-based and retrospective study. We analyzed anonymously all data gathered from the participating institutions.

One hundred and eighty-seven eyes of 187 patients with GON were recruited into this study from five institutions as previously reported [4]. The methods of ophthalmic examination and data collection were described previously [4]. Briefly, presurgical refractive error data were collected from eyes that had undergone refractive procedures including cataract surgery. Data from at least six visual field examinations (approximately every six months) (Carl Zeiss Meditec, Dublin, CA) were also collected retrospectively with the Humphrey visual field analyzer for each patient. MD slope values obtained from those data were divided into two groups: the visual field loss progression group (<−0.3 dB/year) and no-progression group (≧−0.3 dB/year). Additional inclusion criteria included (1) best corrected visual acuity of 0.155 or better (LogMAR); (2) no congenital ONH anomalies; (3) ONH size within the typical normal range, defined as a disc-macula distance-to-disc diameter (DM/DD) ratio of between approximately 2.4 and 3.0; (4) no clinically apparent secondary cause of glaucoma and no other disease affecting the visual field; (5) no history of intraocular surgery other than cataract or glaucoma surgery; (6) no history of cataract or glaucoma surgery in the previous 3 years; and (7) glaucomatous visual field loss of better than −12 dB MD [4]. Other exclusion criteria may include (1) retinal diseases, neuro-ophthalmological diseases, degenerative myopia, and central nervous system diseases which can affect the visual field; (2) pseudoexfoliation; (3) angle closure; and (4) use of systemic or topical steroid. The patients were also queried about a history of systemic hypertension, diabetes mellitus, and hyperlipidemia.

The stereo images of ONH were obtained with a stereo fundus camera (nonmyd WX). The built-in software (VK-2 WX, prototype version, Kowa Company, Ltd., Japan) automatically calculates ONH morphological parameters based on manually set contour lines for the ONH disc and cup as described previously [4]. The disc contour was delineated by the inner margin of Elschnig's scleral ring, and the cup contour was delineated by the outer cup margin, which was indicated by the bending of the ONH vessels at the rim in accordance with the recommendations of the *Japan Glaucoma Society Guideline for Glaucoma*, 3rd edition [4].

### 2.1. Statistical Analysis

Continuous variables were expressed as mean values ± standard deviation. Demographic patient data and ONH parameters were compared between the progression and no-progression groups using the *t*-test for continuous variables and Fisher's exact probability test for categorical variables. Correlations between glaucoma progression and ONH parameters were assessed using stepwise logistic regression analysis in which progression or no progression was set as a dependent variable, and the 38 ONH parameters reported previously [4] were set as independent variables. In the stepwise logistic regression analysis, a forward selection method was used to determine the significant ONH parameter(s). Progression/no progression was fit to the determined ONH parameter(s) using the nominal logistic regression model to calculate the odds ratio of progression. The level of significance was 0.05 in all statistical tests.

## 3. Results

The characteristics of the 187 patients in the visual field progression group (*n* = 50) and no-progression group (*n* = 137) are shown in Table 1. There was no significant difference in the average age and the gender ratio between the progression and no-progression groups (Table 1). There were significantly fewer patients with a history of systemic hypertension in the progression group compared with those in the no-progression group (*p* = 0.037, Table 1), and significantly more used *β*-blocker or carbonic anhydrase inhibitor eyedrops in the former compared with those in the latter group (*p* = 0.029 and *p* = 0.002, resp.). There was also a significant difference in the MD between the progression (−5.88 ± 2.91 dB) and no-progression groups (−4.28 ± 3.28 dB) (*p* = 0.003, Table 1). In addition, there was a significant difference in the PSD between the progression (9.43 ± 3.69 dB) and no-progression (7.59 ± 4.25 dB) groups (*p* = 0.007, Table 1). The averages of MD slopes in the progression group and no-progression group were −0.58 ± 0.28 dB/year and 0.05 ± 0.26 dB/year, respectively (*p* < 0.0001, Table 1).

**Figure 1:**
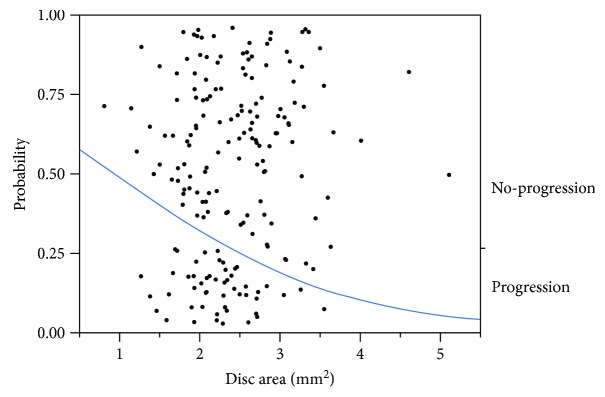
Logistic fit of glaucomatous visual field progression/no progression by disc area. The curve indicates the predicted probability of visual field progression (the left *y*-axis) as a function of disc area (*x*-axis) (*p* = 0.013). In this model, the odds ratio of visual field progression is calculated to be 0.49/mm^2^ disc area.

The ONH parameters in the two groups are shown in Table 2. Among these parameters, vertical disc width (diameter) and disc area in the progression group were significantly less than those in the no-progression group (*p* = 0.017 and *p* = 0.016, resp., Table 2). Unexpectedly, the cup area and cup volume in the progression group were also significantly less than those in the no-progression group (*p* = 0.029 and *p* = 0.04, resp., Table 2). Therefore, further statistical analysis was performed to clarify the association between ONH parameters and visual field progression. Stepwise logistic regression analysis showed that among the 38 ONH parameters, only the disc area was selected as a significant factor associated with visual field progression (*p* = 0.013). In the nominal logistic regression model (Figure 1), the odds ratio of visual field loss progression was calculated to be 0.49/mm^2^ disc area.

## 4. Discussion

The present study found that a history of systemic hypertension is not a risk factor for visual field progression, since fewer patients in the progression group had systemic hypertension. Consistent with this result, previous studies showed that a lower diastolic blood pressure was associated with glaucomatous progression [8, 9]. Comparisons of other characteristics demonstrated that the use of *β*-blocker or carbonic anhydrase inhibitor eyedrops was more common in the visual field progression group. It is reasonable to assume that prostaglandin (PG) eyedrops are the first choice for administration in glaucoma patients, and therefore, PG alone is not associated with disease progression. However, the administration of multiple types of eyedrops may be associated with progression.

We also found significantly worse MD and PSD values in the progression group compared with the no-progression group. This is consistent with a previous review article demonstrating that the baseline MD was significantly associated with the incidence of progression, with the incidence rate increasing by 0.9 percentage points per additional dB of MD loss [10]. It is also interesting to note previous findings showing that the rate of progression increased with the severity of visual field loss (*β* = 0.18; *p* = 0.001) in patients with mild visual field loss (MD > −6 dB) but did not increase in the same manner in patients with moderate (MD≦−6 to ≧−12 dB) or severe loss (MD < −12 dB) [11]. That study suggested that in the most severe cases, visual field worsening cannot be observed as the field gets closer to total perimetric blindness [11]. Because we excluded patients with severe visual field loss worse than −12 dB MD, the average MD in the 187 patients included in this study was −4.71 ± 3.26 dB, meaning that 68% could be categorized as having mild field loss [4].

In the nominal logistic regression model (Figure 1), the smallest disc exists in the no-progression group (the right *y*-axis); therefore, it seems that merely the disc size cannot distinguish the visual field progression group from the no visual field progression group. In fact, there are several controversial reports regarding the disc size and glaucomatous damage. For example, some reports suggested that a large disc is a risk factor and the stress to the lamina cribrosa is greater in eyes with a larger disc than those with a smaller disc [12]. In contrast, other studies suggested that larger discs were more likely to be classified as glaucomatous whether they were glaucomatous or not, while small discs were more likely to be classified as normal [13, 14]. Moreover, two different groups reported that the retinal nerve fiber layer (RNFL) thickness significantly increased with a greater optic disc size [15, 16], although care must be taken in interpreting the relationship between disc size and RNFL thickness found in optical coherence tomography studies [6]. Taken together with the finding that the optic nerve fiber count increased with greater disc size in a human histological study [7], it is possible that smaller optic discs may contain fewer optic nerve axons than larger ones. Therefore, one hypothesis is that smaller optic discs may be more vulnerable than larger discs. Consistent with this hypothesis, we found that the vertical disc width and disc area in the visual field progression group were significantly smaller than those in the no-progression group. However, cup area and cup volume in the progression group were also smaller than those in the no-progression group. Because the vertical (0.83 ± 0.08 versus 0.82 ± 0.08) and horizontal C/D ratios (0.73 ± 0.08 versus 0.75 ± 0.09) did not differ between the two groups in the current study, smaller cup area and cup volume may be the result of smaller disc size in the progression group. Moreover, it is interesting to note that the results of a recent study indicated that the C/D ratio is smaller for small discs and positively correlated with the disc size in healthy human eyes [17]. In the present study, the curve in the nominal logistic regression model (Figure 1) indicates the predicted probability of visual field progression (the left *y*-axis), implicating that larger discs may have lower probability. Our current logistic regression analysis revealed a significant association between visual field loss progression and disc area (odds ratio 0.49/mm^2^ disc area), indicating that if the disc area increased by 1 mm^2^, the probability of progression would decrease by about half. This also means that if the disc area decreased by 1 mm^2^, the probability of progression would be approximately twofold greater.

This study had several limitations. It was retrospective and hospital based. From a technical viewpoint, in some cases such as those with temporal peripapillary atrophy or shallow cupping, it can be difficult to define the margins accurately. Moreover, it is possible that the amount of rim width asymmetry is an important factor which may affect disc size results, although the rim decentering, which may reflect with asymmetry, did not differ significantly between the progression group and no-progression group. In addition, although the typical normal ONH size was defined as a DM/DD ratio of between approximately 2.4 and 3.0, both the disc size and the DM distance may increase in some cases such as high myopia. However, most cases included in the present study were within the typical normal range, and therefore, it remains to be elucidated whether smaller or larger discs also fit this concept. Nonetheless, taking the results of the present phase of the GSAS together, these findings suggest that a smaller disc area may be associated with more rapid glaucomatous visual field progression.

## Figures and Tables

**Table 1:** Comparison of demographic data between patients in the progression and no-progression groups.

Patient data	Progression (*n* = 50)	No progression (*n* = 137)	*p* Value
Age (years)	61.48 ± 8.92	61.33 ± 9.58	0.92
Sex (male : female)	25 : 25	62 : 75	0.62
Hypertension (+)	7 (14%)	41 (29.93%)	0.037
Corneal curvature radius on the test day (mm)	7.65 ± 0.25	7.68 ± 0.28	0.52
Pretreatment IOP (mmHg)	16.97 ± 4.87	16.92 ± 4.09	0.94
PG (+)	35 (70%)	93 (67.88%)	0.86
*β*-Blocker (+)	28 (56%)	51 (37.23%)	0.029
CAI (+)	16 (32%)	16 (11.68%)	0.002
Antiglaucoma eyedrops (number)	1.70 ± 1.04	1.20 ± 0.88	0.0012
MD (dB)	−5.88 ± 2.91	−4.28 ± 3.28	0.0028
PSD (dB)	9.43 ± 3.69	7.59 ± 4.25	0.0074
Pretreatment spherical equivalent refractive error (D)	−3.49 ± 3.89	−3.34 ± 3.72	0.8
MD slope (dB/year)	−0.58 ± 0.28	0.05 ± 0.26	<0.0001

PG = prostaglandin; CAI = carbonic anhydrase inhibitor; MD = mean deviation; PSD = pattern standard deviation.

**Table 2:** Comparison of ONH parameters between the progression and no-progression groups.

Optic nerve head parameters	Progression (*n* = 50)	No-progression (*n* = 137)	*p* Value
Vertical disc width	1.79 ± 0.18	1.89 ± 0.24	0.017
Horizontal disc width	1.60 ± 0.24	1.69 ± 0.29	0.052
Vertical cup-disc ratio	0.83 ± 0.08	0.82 ± 0.08	0.574
Horizontal cup-disc ratio	0.73 ± 0.08	0.75 ± 0.09	0.227
Minimum rim-disc ratio	0.018 ± 0.024	0.018 ± 0.025	0.979
Superior minimum rim-disc ratio	0.077 ± 0.055	0.086 ± 0.060	0.363
Inferior minimum rim-disc ratio	0.043 ± 0.060	0.033 ± 0.040	0.209
Superior rim width	0.19 ± 0.09	0.21 ± 0.10	0.161
Inferior rim width	0.12 ± 0.11	0.12 ± 0.09	0.922
Mean cup depth	0.19 ± 0.07	0.21 ± 0.09	0.202
Maximum cup depth	0.50 ± 0.15	0.53 ± 0.20	0.368
Height variation contour	0.57 ± 0.22	0.58 ± 0.28	0.738
Disc area	2.24 ± 0.46	2.49 ± 0.67	0.016
Cup area	1.30 ± 0.47	1.50 ± 0.59	0.029
Cup-disc area ratio	0.57 ± 0.12	0.59 ± 0.11	0.265
Rim area	0.94 ± 0.26	0.99 ± 0.27	0.301
Rim-disc area ratio	0.43 ± 0.12	0.41 ± 0.11	0.265
Rim-disc ratio of section 1	0.076 ± 0.045	0.067 ± 0.046	0.204
Rim-disc ratio of section 2	0.11 ± 0.05	0.11 ± 0.06	0.94
Rim-disc ratio of section 3	0.18 ± 0.06	0.17 ± 0.06	0.257
Rim-disc ratio of section 4	0.20 ± 0.08	0.19 ± 0.07	0.462
Rim-disc ratio of section 5	0.15 ± 0.09	0.14 ± 0.08	0.568
Rim-disc ratio of section 6	0.065 ± 0.061	0.056 ± 0.045	0.293
Disc volume	0.87 ± 0.29	0.96 ± 0.47	0.217
Cup volume	0.25 ± 0.14	0.33 ± 0.24	0.04
Rim volume	0.17 ± 0.09	0.17 ± 0.10	0.995
Rim decentering	0.28 ± 0.44	0.31 ± 0.42	0.668
Disc tilt angle	11.51 ± 12.97	10.07 ± 12.29	0.486
